# Circular RNA-Expression Profiling Reveals a Potential Role of Hsa_circ_0097435 in Heart Failure via Sponging Multiple MicroRNAs

**DOI:** 10.3389/fgene.2020.00212

**Published:** 2020-03-10

**Authors:** Jiaqi Han, Liwei Zhang, Longgang Hu, Hua Yu, Fengqiang Xu, Bin Yang, Rui Zhang, Yongtao Zhang, Yi An

**Affiliations:** ^1^Department of Medicine, Qingdao University, Qingdao, China; ^2^Affiliated Cardiovascular Hospital of Qingdao University, Qingdao University, Qingdao, China; ^3^Affiliated Hospital of Qingdao University, Qingdao University, Qingdao, China

**Keywords:** circular RNA, heart failure, miRNA sponge, NGS – next generation sequencing, cardiomyocyte apoptosis

## Abstract

Circular RNAs represent a new type of non-coding RNA molecules that influence the occurrence and development of various human diseases by sponging microRNAs, although their roles in heart failure have not been clarified. In this study, peripheral blood samples from 5 patients with heart failure and 4 healthy volunteers were analyzed by next-generation sequencing (NGS) to screen for differentially expressed Circular RNAs. Fifty-six differentially expressed Circular RNAs were identified, of which 29 were up-regulated and 27 were down-regulated. Dysregulated expression of 6 Circular RNAs was verified by quantitative polymerase chain reaction (PCR) analysis, and hsa_circ_0097435 expression was confirmed to be significantly up-regulated in 40 patients with heart failure. Further study with extracted exosomes showed that hsa_circ_0097435 expression was significantly higher in patients with heart failure. In cardiomyocytes, hsa_circ_0097435 was up-regulated after doxorubicin treatment, promoting cardiomyocyte apoptosis. Hsa_circ_0097435 overexpression promoted cardiomyocyte apoptosis, and silencing hsa_circ_0097435 inhibited apoptosis. Moreover, RNA-pulldown experiments and AGO2-immunoprecipitation experiments revealed that hsa_circ_0097435 potentially served a role in heart failure by sponging multiple microRNAs. Collectively, these results suggest that hsa_circ_0097435 can be used as a biological blood marker and revealed a new pathway involved in regulating myocardial cell injury. Our findings may provide a rational basis for developing new treatments for heart failure.

## Introduction

Almost all cardiovascular diseases eventually lead to heart failure (HF). Despite advances in clinical and drug interventions that have increased the survival time of patients with HF, mortality rates remain high (Targher et al., 2017). Recent research has provided guidance for the diagnosis, treatment, and prognosis of HF. Circulating blood biomarkers are becoming increasingly important in daily clinical practice because of the high sensitivity and accuracy of the associated non-invasive methods (Devaux et al., 2017). Natriuretic peptides, especially b-type natriuretic peptide (BNP), are blood biomarkers that are frequently measured for HF-related diagnosis and prognosis, but are not applicable to all types of heart failure (Brunner-La Rocca et al., 2015). Therefore, a new blood biomarker is needed to better diagnose, treat, and predict the prognosis of HF.

Circular RNA (circRNA) molecules have a stable, closed-ring structure and are widely present in whole blood, plasma, and extracellular vesicles (Koh et al., 2014; Li P. et al., 2015; Vausort et al., 2016). CircRNAs are regulated during cardiac development and failure (Khan et al., 2016; Werfel et al., 2016), so they offer great potential as HF biomarkers. Previous findings have revealed various circRNAs that were deregulated during HF caused by myocardial infarction in mice, which suggests a potential role of circRNAs in HF (Wu et al., 2016). However, little is known about the expression profiles and functions of circRNAs in HF. CircRNA molecules are rich in microRNA (miRNA)-binding sites and act as an miRNA sponge in cells, thereby removing the inhibitory effects of miRNAs on their target genes and increasing the target gene-expression levels. This mechanism is known as the competitive endogenous RNA (ceRNA) mechanism. A recent study revealed a novel regulatory pathway comprising circNCX1, miR-133a-3p, and CDIP1, which was found to be involved in cardiomyocyte apoptosis (Li et al., 2018). This pathway may serve as a therapeutic avenue for ischemic heart diseases. Apoptosis has important structural and functional effects on heart failure. The study of apoptosis is helpful for the treatment and prevention of heart failure. Understanding the mechanism of cardiac apoptosis and the mechanism of ceRNA is of great significance for preventing cardiac injury and treating HF. Exosomes are small membranous vesicles that can be isolated from serum and urine (Bae et al., 2018). It has been reported that plasma circRNAs can be encapsulated in exosomes (Dou et al., 2016; Tang et al., 2018). In HF, the expression of circRNAs in plasma exosomes also needs to be further studied.

In this study, next-generation sequencing (NGS) was performed to identify differentially expressed circRNAs between HF and healthy control subjects. We aimed to identify circRNA profiles in peripheral blood cells from patients with HF and to explore the roles of circRNAs in HF pathogenesis. We found that hsa_circ_0097435 expression was markedly increased in patients with HF. Further study showed that the level of hsa_circ_0097435 in exosomes from patients with HF was significantly increased. In cardiomyocytes, hsa_circ_0097435 was up-regulated after doxorubicin (DOX) treatment, promoting apoptosis in cardiomyocytes. The expression level of hsa_circ_0097435 participates in regulating apoptosis. Hsa_circ_0097435 overexpression promoted apoptosis in cardiomyocytes, and silencing hsa_circ_0097435 expression inhibited apoptosis in cardiomyocytes. Hsa_circ_0097435 overexpression resulted in significant increases in the recovery of 5 miRNAs in RNA-pulldown assays, and the AGO2 protein was significantly pulled down by hsa_circ_0097435. Immunoprecipitation of AGO2 from AC16 cells showed that AGO2 protein directly bound to 4 miRNAs. Therefore, we predicted that hsa_circ_0097435 could bind to miRNAs through the AGO2 protein and act as a sponge for multiple miRNAs.

## Materials and Methods

### Collection of Samples

Forty-five patients with HF and 44 healthy volunteers who underwent physical examinations in the Affiliated Hospital of Qingdao University were included in this study, and informed consent forms were signed by all participants. Among the patient samples, we selected 5 from patients with HF and 4 from volunteers for sequencing. The inclusion criteria were clinical symptoms, b-mode ultrasound results, and laboratory examinations. Routine blood-examination results from all participants were within the normal range. Six milliliters of Trizol reagent (M5 Liquid Sample Total RNA Extraction Reagent, Mei5 Bio, Beijing, China) was added to each 2 mL peripheral blood sample immediately after collection, and the samples were preserved at –80°C for subsequent RNA extraction.

### RNA Quantification and Qualification

RNA degradation and contamination, especially DNA contamination, was monitored on 1.5% agarose gels. RNA concentrations and purities were measured using a NanoDrop 2000 Spectrophotometer (Thermo Fisher Scientific, Wilmington, DE, United States). RNA integrity was assessed using the RNA Nano 6000 Assay Kit and the Agilent Bioanalyzer 2100 System (Agilent Technologies, CA, United States). The optical density (OD) ratio (absorbance at 260 nm divided by that at 280 nm) of pure RNA ranged between 1.8 and 2.1. All quality standards set by the manufacturer were met.

### Library Preparation for CircRNA Sequencing

For each sample, 1.5 μg RNA was used as input material for rRNA removal using the Ribo-Zero rRNA Removal Kit (Epicentre, Madison, WI, United States), followed by digestion of linear RNA using RNase R. Sequencing libraries were generated using the NEBNext^®^ Ultra^TM^ Directional RNA Library Prep Kit for Illumina^®^ [New England BioLabs (NEB), United States] following the manufacturer’s recommendations, and index codes were added to attribute the sequences to each sample. Briefly, fragmentation was performed using divalent cations at an elevated temperature in NEBNext First Strand Synthesis Reaction Buffer (5×). First-strand complementary DNA (cDNA) was synthesized using random hexamer primers and reverse transcriptase. Second-strand cDNA synthesis was subsequently performed using DNA Polymerase I and RNase H. The remaining overhangs were converted into blunt ends via exonuclease/polymerase activities. After adenylation of the 3′ ends of the DNA fragments, NEBNext Adaptors with a hairpin loop structure were ligated to prepare for hybridization. To select for insertion fragments, preferentially 150–200 base pairs in length, the library fragments were purified using AMPure XP Beads (Beckman Coulter, Beverly, MA, United States). Then, the cDNA fragments were treated with 3 μL USER Enzyme (NEB, United States) at 37°C for 15 min before amplification by polymerase chain reaction (PCR). PCR was performed with Phusion High-Fidelity DNA polymerase, Universal PCR primers, and the Index(X) Primer. Finally, the PCR products were purified (AMPure XP system) and the quality of each library was assessed on an Agilent Bioanalyzer 2100 and by performing quantitative PCR (qPCR).

### Clustering and Sequencing

Clustering of the index-coded samples was performed on a cBot Cluster Generation System using TruSeq PE Cluster Kit v3-cBot-HS (Illumina), according to the manufacturer’s instructions. After cluster generation, the prepared libraries were sequenced using an Illumina platform, and reads were generated.

### CircRNA-Profiling Analysis

CircRNAs were predicted based on the number of junction reads identified using the CircRNA Identifier (CIRI) tool and find_circ software. Differential-expression analysis of two conditions/groups was performed using the DESeq R package. DESeq provides statistical routines for determining differential expression in digital gene-expression data using a model based on the negative binomial distribution. The resulting *P*-values were adjusted using the Benjamini–Hochberg approach for controlling the false-discovery rate. Genes with an adjusted *P* < 0.01 and an absolute log_2_ (fold-change) > 1 found using DESeq were assigned as being differentially expressed.

### Plasma Exosome Extraction

All exosome-extraction steps were performed following the instructions of the Hieff^TM^ Quick Exosome Isolation Kit (for Serum/Plasma; Yeasen Biotechnology, Co., Ltd., Shanghai, China). Blood samples (1 mL each) were transferred to individual centrifuge tubes and centrifuged for 10 min at 3000 × *g* and 4°C, after which the particles were discarded and each supernatant was transferred to a new centrifuge tube. After pretreatment, 4 volumes of 1× phosphate-buffered saline were added to each tube, and the samples were mixed evenly.

### Validation by Real-Time qPCR

Six upregulated circRNAs found in blood cells from 40 HF patients and 40 healthy controls were verified by real-time qPCR. Total RNA was used for synthesizing cDNAs with the PrimeScript RT Reagent Kit (Takara, Tokyo, Japan). First-strand cDNA (2 mL) was used for PCR experiments (performed in triplicate), with TB Green Premix Ex Taq II (Takara, Tokyo, Japan). Beta-actin was detected as an internal reference for circRNAs to avoid potential aberrances in concentrations and transcription efficiencies. Relative circRNA-expression levels were measured using the 2^–ΔΔCT^ method for each circRNA. The relative expression of miRNAs was calculated using the same real-time qPCR method as verified with circRNAs. U6 RNA was detected as an endogenous control gene for miRNAs.

### Construction of Overexpression Vectors

Hsa_circ_0097435 and hsa_circ_0097435-MS2 were synthesized by total gene synthesis, and cloned individually into the pLC5-ciR vector using the *Eco*RI and *Bam*HI restriction enzyme sites. The chromatograms were normal, without any hybrid peaks or superimposed bands, and the sequence comparison was consistent, indicating that hsa_circ_0097435 and hsa_circ_0097435-MS2 were successfully inserted into pLC5-ciR, and the overexpression vectors were successfully constructed. The overexpressed plasmids and the parental vector were separately transfected into ac16 cells using the Lipofectamine^TM^ 3000 transfection reagent (Thermo Fisher Scientific, Waltham, MA, United States), and overexpression of the target gene was measured by qPCR.

### SiRNA Knockdown

Small-interfering RNA (siRNA) oligonucleotides specific for hsa_circ_0097435 were designed using Ambion’s siRNA design tool and purchased from GenePharma Co., Ltd. (Shanghai, China). The siRNA sequence for hsa_circ_0097435 was 5′-ACUUGUGAUGCUGACUUGGTTCCAAGUCAGCAUCA CAAGUTT-3′. The specificity of the oligonucleotides was confirmed through comparing with all other sequences in GenBank using Nucleotide BLAST. Transfection of siRNAs was performed using the Lipofectamine^TM^ 3000 transfection reagent (Thermo Fisher Scientific), according to the manufacturer’s instructions.

### Apoptosis Assays

Apoptosis was determined by performing terminal deoxynucleotidyl transferase dUTP nick-end labeling (TUNEL) assay using the TUNEL Apoptosis Detection Kit (Yeasen Biotechnology, Co., Ltd., Shanghai, China), per the manufacturer’s instructions. The samples were stained with anti-fluorescence attenuation sealant containing DAPI (Solarbio, Beijing, China) and detected with a Zeiss LSM510 META microscope. The percentage of apoptotic nuclei was calculated by dividing the total number of TUNEL-positive nuclei by the total number of DAPI-positive nuclei.

### Prediction of miRNAs Association With Hsa_circ_0097435 and Their Target mRNAs

Go to http://circinteractome.nia.nih.gov. Select the “miRNA Target Sites” tab. CircInteractome (Panda et al., 2018) uses the TargetScan to predict the miRNAs which have sequence complementarity with hsa_circ_0097435. Furthermore, miRNAs associated with hsa_circ_0097435 were identified using circMir program (purchased from http://www.bioinf.com.cn/) that uses miRanda (Betel et al., 2008) and RNAhybrid (Rehmsmeier et al., 2004) to predict miRNAs. Finally, miRNAs predicted by at least two softwares including TargetScan, miRanda and RNAhybrid were selected. Targetscan (Lewis et al., 2003) and miRanda were then used to predict the target mRNAs of miRNAs.

### Pulldown Assays

MS2 is a tag sequence that can specifically bind to the capture protein, MS2-CP. The MS2 labeling system was used to express hsa_circ_0097435-MS2 in cells. The highly specific and stable interaction between hsa_circ_0097435-MS2 and the captured protein MS2-CP was employed to capture hsa_circ_0097435 and its interacting molecules. We constructed both an hsa_circ_0097435-overexpression plasmid (C97435) and an hsa_circ_0097435-MS2-overexpression plasmid (C97435-MS2). The expression vectors C97435-MS2 and MS2-CP were transfected in the cells to induce transient MS2-CP expression. MS2-CP and MS2-labeled circRNAs could specifically bind to form an MS2-CP-MS2–circRNA complex. The MS2-CP-MS2–circRNA complex was pulled down, and the capture products were detected to identify proteins or miRNA molecules that may interact with hsa_circ_0097435.

### AGO2 Immunoprecipitation

An AGO2-specific antibody was used for AGO2 immunoprecipitation, and an IgG antibody was selected as the negative control. Mouse monoclonal anti-AGO2 (Abcam, Cambridge, England) or mouse normal IgG antibody (Abcam, Cambridge, England) were preincubated with Magna Bind goat anti-mouse IgG Magnetic Bead slurry (Thermo Fisher Scientific, Waltham, MA, United States) and used for immunoprecipitation. In brief, cells were lysed in 150 mM KCl, 25 mM Tris-HCl, pH 7.4, 5 mM EDTA, 0.5% Triton X-100, and 5 mM DTT supplemented with RNase inhibitor (Takara, Tokyo, Japan) and proteinase inhibitor cocktail (Roche Applied Science, Basel, Switzerland). The lysate was mixed with antibody-coupled Sepharose beads and left under rotation for 4 h at 4°C. Beads were subsequently washed six times in lysis buffer and the RNA was extracted using Trizol reagent(Takara, Tokyo, Japan).

### GO and KEGG Analysis

The Gene Ontology (GO) database is a structured standard biological annotation system established by the GO Consortium in 2000, which is aimed at establishing a standard vocabulary of knowledge regarding genes and their products that is applicable to various species. GO function classification statistics of the mRNAs targeted by the miRNAs associated with hsa_circ_0097435 was implemented using the clusterProfiler R packages. Kyoto Encyclopedia of Genes and Genomes (KEGG) is a database resource for understanding the high-level functions and utilities of biological systems, such as a cell, an organism, or an ecosystem, based on molecular-level information, especially large-scale molecular datasets generated by genome sequencing and other high-throughput experimental technologies^1^. The clusterProfiler R packages were used to identify which KEGG pathways the target genes were significantly enriched in.

### Statistical Analysis

Data are expressed as mean ± standard error of the mean of at least three independent experiments. One-way analysis of variance was used for multiple comparisons. Student’s unpaired *t*-test was used for comparisons of two groups. A *p* < 0.05 was considered to reflect a statistically significant difference (^∗^*p* < 0.05, ^∗∗^*p* < 0.01, ^∗∗∗^*p* < 0.001). GraphPad Prism software, version 8 (GraphPad Software, San Diego, CA, United States) was applied for data management and analysis.

## Results

### CircRNA Analysis

**FIGURE 1:**
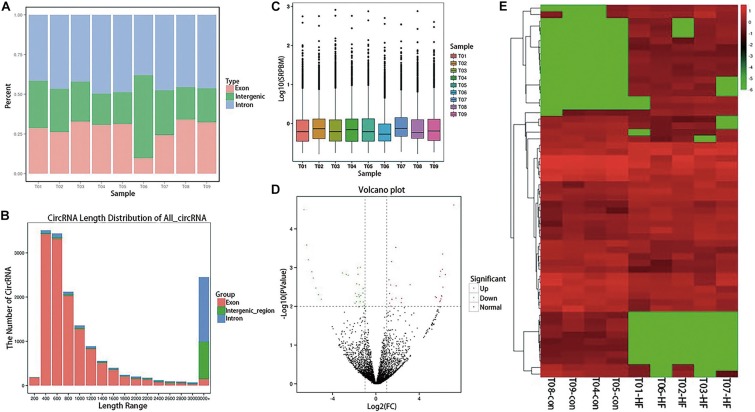
Expression profiling of circRNAs in patients with HF. **(A)** A histogram of read distributions in different regions of the genome. Each column represents a sample, and the height of the region represents the percentage of all mapped reads mapping to that region. **(B)** CircRNA length-distribution map. The horizontal coordinate represents the length interval of circRNA. The ordinate represents the number of circRNAs in the length interval. **(C)** SRPBM boxplot of each sample. The expression of circRNA in each sample was counted and normalized by SRPBM algorithm. In the figure, the abscissa represents different samples, and the ordinate represents the logarithm value of normalized sample expression quantity **(D)** Differential-expression volcano diagram. Each point in the differential-expression volcano diagram represents a circRNA, and the abscissa represents the log value of the differential multiple of a certain circRNA in the two samples. The y-coordinate represents the negative log of the *p*-value. In the figure, the green data points represent down-regulated differentially expressed circRNAs, the red data points represent up-regulated differentially expressed circRNA, and the black data points represent non-differentially expressed circRNAs. **(E)** CircRNA cluster heatmap with differential expression. The abscissa represents the sample name and the clustering result for each sample, whereas the ordinate represents different circRNAs and the clustering results for the circRNAs. The different columns in the figure represent different samples, and the different rows represent different circRNAs. The color represents the log_10_ of the circRNA-expression level in each sample.

After sequencing quality control, 102.45 gb of clean data was obtained, and the percentage of Q30 bases in each sample was not less than 95.16%. RNA sequencing data was deposited in the NCBI Sequence Read Archive (BioProject: PRJNA574863). After obtaining clean reads, the sequences were aligned with the reference genome to determine their locations within the reference genome or gene, as well as the sequence characteristics unique to the sequencing samples. Alignments were performed using the Burrows–Wheeler Aligner (Gao et al., 2015). Reads that were mapped to a specified reference genome are referred to as mapped reads, and the corresponding data are referred to as mapped data. The numbers of mapped reads in different regions (exons, introns, and intergenomic regions) of the specified reference genome were counted, and a mapped reads-distribution histogram was generated for each sample with different regions of the genome (Figure 1A). The CIRI (Supplementary Table S1) and find_circ (Supplementary Table S2) were used to predict circRNA, and the intersection of the two software prediction results was taken. Distribution statistics were conducted for all circRNA lengths identified (Figure 1B). The circRNA-expression levels in each sample were statistically analyzed. Not only could differences in the expression-level distributions be seen in individual samples, but the overall expression level of different samples could be intuitively compared (Figure 1C).

### Expression Profiling of CircRNAs in Patients With HF

Differential-expression analysis between the HF and normal control (NC) groups was performed using the DESeq R package. DESeq provides statistical routines for determining differential expression in digital gene-expression data using a model based on the negative binomial distribution. The resulting *P*-values were adjusted using the Benjamini–Hochberg approach for controlling the false-discovery rate. Genes with an adjusted *P* < 0.01 and an absolute log_2_ (fold-change) > 1 found by DESeq were considered to be differentially expressed. Finally, 56 circRNAs with significant differences in expression were identified (Supplementary Table S3). Compared with the NC group, 29 circRNAs in the HF group were significantly up-regulated and 27 were significantly down-regulated, as shown in the volcano diagram (Figure 1D) and cluster heatmap (Figure 1E).

### Verification of Selected CircRNAs

**FIGURE 2:**
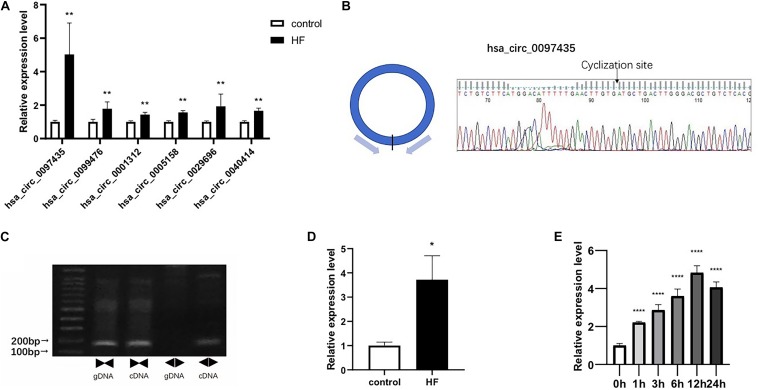
Verification of selected circRNAs. **(A)** Six circRNAs were upregulated in the blood of patients with HF compared to the NC group. These six circRNAs included hsa_circ_0097435 (**p < 0.01), hsa_circ_0099476 (**p < 0.01), hsa_circ_0001312 (**p < 0.01), hsa_circ_0005158 (**p < 0.01), hsa_circ_0029696 (**p < 0.01), and hsa_circ_0040414 (**p < 0.01). **(B)** The amplification products of hsa_circ_0097435 were sequenced by Sanger sequencing to verify the cyclization site and ring-formation mode of hsa_circ_0097435. **(C)** Use of the divergent-primer strategy to verify the circular structure of hsa_circ_0097435. **(D)** The levels of hsa_circ_0097435 in exosomes of patients with HF was significantly higher than those in normal volunteer subjects. The hsa_circ_0097435 level was analyzed by qRT-PCR. *P < 0.05 versus control. *n* = 15. **(E)** AC16 cells were treated with 2 μM DOX. Total RNA was isolated and reverse-transcribed. The hsa_circ_0097435 level was analyzed by qRT-PCR. ****P < 0.0001 versus 0 h. *n* = 3.

Real-time qPCR was used to verify the peripheral circRNAs of 40 patients with HF and 40 healthy volunteers. For further study, we focused on upregulated circRNAs with certain circBase IDs, and selected 6 circRNAs for verification with RT-PCR. The primer sequences were detailed in Supplementary Table S4. The levels of hsa_circ_0097435 (*p* < 0.01), hsa_circ_0099476 (*p* < 0.01), hsa_circ_0001312 (*p* < 0.01), hsa_circ_0005158 (*p* < 0.01), hsa_circ_0029696 (*p* < 0.01), and hsa_circ_0040414 (*p* < 0.01) were significantly higher in the HF group than in the NC group, and the expression difference of hsa_circ_0097435 was most obvious (Figure 2A).

We detected the expression of hsa_circ_0097435 as a circRNA by amplifying it from the cDNA of AC16 cardiomyocytes, using divergent primers (Figure 2C and Supplementary Figure S1). In addition, sequencing the amplification product of hsa_circ_0097435 by Sanger sequencing was used to verify the circular connection of circRNA (Figure 2B). To verify the differential expression of hsa_circ_0097435 in HF, plasma exosomes of 15 patients with HF and 15 healthy volunteers were extracted to detect the hsa_circ_0097435 levels in exosomes. Significantly higher levels of hsa_circ_0097435 (*p* < 0.05) were detected in exosomes from patients with HF than in those from normal volunteers (Figure 2D). To understand the role of hsa_circ_0097435 in cardiomyocyte apoptosis, we further examined its production in DOX-treated cardiomyocytes. A time-dependent increase in hsa_circ_0097435 levels was observed in AC16 cells following treatment with 2 μM DOX, which peaked at 12 h after DOX treatment (Figure 2E). Therefore, the level of hsa_circ_0097435 increased after myocardial cell injury.

### Hsa_circ_0097435 Promoted Apoptosis in Cardiomyocytes

**FIGURE 3:**
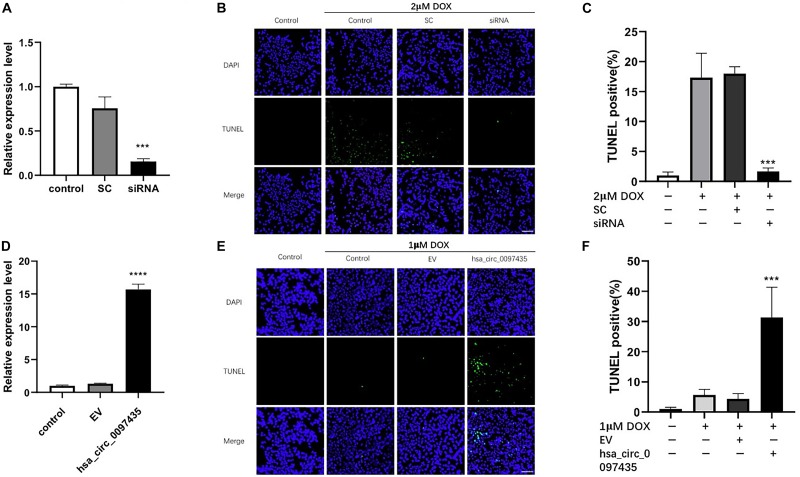
hsa_circ_0097435 promoted apoptosis in cardiomyocytes. **(A)** AC16 cells were transfected with an siRNA vector targeting hsa_circ_0097435. The expression level of hsa_circ_0097435 was detected by qRT-PCR. SC, scramble control; siRNA, siRNA targeting the junction region in hsa_circ_0097435. ***P < 0.001 versus SC. *n* = 3. **(B)** AC16 cells were transfected with an hsa_circ_0097435 siRNA-expression vector and treated with 2 μM DOX for 12 h. Apoptosis was evaluated by performing TUNEL assays. Green, TUNEL-positive nuclei; blue, DAPI-stained nuclei. Scale bars, 100 μm. **(C)** The apoptosis rate calculated from three independent experiments. ***P < 0.001 versus SC. *n* = 3. **(D)** AC16 cells were transfected with an hsa_circ_0097435-overexpression vector. The expression level of hsa_circ_0097435 was detected by qRT-PCR. EV: empty vector. ****P < 0.0001 versus EV. *n* = 3. **(E)** AC16 cells were transfected with the hsa_circ_0097435-overexpression vector and treated with 1 μM DOX for 12 h. Apoptosis was detected by performing TUNEL assays. Green, TUNEL-positive nuclei; blue, DAPI-stained nuclei. Scale bars, 100 μm. **(F)** The rate of apoptosis was calculated using data from three independent experiments. ***P < 0.001 versus EV. *n* = 3.

To verify whether the increased hsa_circ_0097435 level was related to myocardial apoptosis, we conducted a series of validation analyses using loss-of-function and gain-of-function experiments. Specifically targeting siRNA at the junction region of hsa_circ_0097435 was used to down-regulate hsa_circ_0097435 expression in AC16 cells (Figure 3A). Silencing hsa_circ_0097435 effectively inhibited DOX-induced apoptosis in AC16 cells (Figures 3B,C). Next, we successfully overexpressed hsa_circ_0097435 in AC16 cells (Figure 3D). We found that AC16 cells treated with a low DOX concentration only showed a slight induction of apoptosis, whereas AC16 cells were more sensitive to DOX treatment after hsa_circ_0097435 overexpression (Figures 3E,F). In summary, these results suggest that hsa_circ_0097435 promoted apoptosis in cardiomyocytes.

### Hsa_circ_0097435 Acted as a Sponge for Multiple miRNAs

**FIGURE 4:**
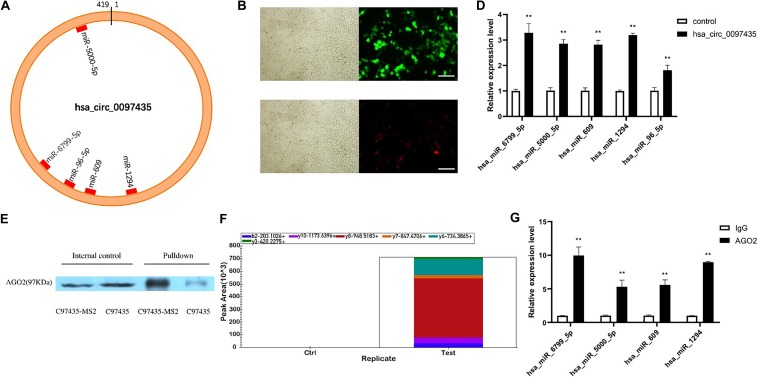
Hsa_circ_0097435 acted as a sponge for multiple miRNAs. **(A)** The potential binding sites of miRNAs related to hsa_circ_0097435 are shown. **(B)** Detection of transient, efficient expression of C97435-MS2 (green fluorescence), Scale bars, 100 μm. **(C)** Detection of efficient expression of MS2-CP (red fluorescence), Scale bars, 100 μm. **(D)** Compared with the negative-control group, 5 miRNAs in the circRNA-enrichment group were upregulated, including hsa_miR_6799_5P (**p < 0.01), hsa_miR_5000_5P (**p < 0.01), hsa_miR_609 (**p < 0.01), hsa_miR_1294 (**p < 0.01), and hsa_miR_96_5P (**p < 0.01). **(E)** WB analysis of pulldown products. The AGO2 protein was significantly pulled down by hsa_circ_0097435. The amount of AGO2 protein in the C97435-MS2 pulldown group was significantly higher than in the C97435 pulldown group. **(F)** Histogram showing quantitative comparison of the polypeptide TTPQTLSNLCLK between the sample groups. AGO2 immunoprecipitation was followed by mass spectrometry, and 3–8 fragment ions of the specific peptide TTPQTLSNLCLK were selected for quantitative analysis. **(G)** Immunoprecipitation of AGO2 from AC16 cells. Compared with the IgG group, 4 miRNAs in the AGO2 group were upregulated, including hsa_miR_6799_5P (**p < 0.01), hsa_miR_5000_5P (**p < 0.01), hsa_miR_609 (**p < 0.01), and hsa_miR_1294 (**p < 0.01).

CircRNA plays important regulatory roles in diseases by interacting with disease-related miRNAs. Based on the gene-sequence information of hsa_circ_0097435, miRNA predictions were performed using CircInteractome, miRanda and RNAhybrid (Supplementary Table S5). CircInteractome uses the TargetScan to predict the miRNAs which have sequence complementarity with hsa_circ_0097435. Finally, 5 miRNAs predicted by at least two softwares including TargetScan, miRanda and RNAhybrid were selected (Supplementary Table S6). Binding sites of these miRNAs in the hsa_circ_0097435 region were found (Figure 4A). To verify that hsa_circ_0097435 acts as a miRNA sponge in cells, we conducted circRNA-pulldown experiments. We constructed both an hsa_circ_0097435-overexpression plasmid (C97435) and an hsa_circ_0097435-MS2-overexpression plasmid (C97435-MS2). After transfection, the transfection efficiency of C97435-MS2 (Figure 4B) and the expression efficiency of MS2-CP (Figure 4C) were detected. MS2 is a tag sequence that can specifically bind to the captured protein, MS2-CP. The expression vectors C97435-MS2 and MS2-CP were transfected in the cells to induce transient MS2-CP expression. MS2-CP and MS2-labeled circRNAs could specifically bind to form an MS2-CP-MS2–circRNA complex. The MS2-CP-MS2–circRNA complex was pulled down, and the target miRNAs were detected by RT-qPCR (Figure 4D). The experimental results demonstrated that these miRNAs could indeed bind to hsa_circ_0097435.

To verify the binding mode between hsa_circ_0097435 and miRNAs, we conducted western blot (WB) experiments on the pulled-down products, which showed that AGO2 protein was significantly pulled down by hsa_circ_0097435 (Figure 4E and Supplementary Figure S2). Further, we conducted AGO2-immunoprecipitation experiments. The AGO2 protein was analyzed by mass spectrometry (Figure 4F). Immunoprecipitation of AGO2 from AC16 cells showed that AGO2 protein directly bound to 4 miRNAs (Figure 4G). Therefore, we predicted that hsa_circ_0097435 could bind to miRNAs through the AGO2 protein and act as a sponge for multiple miRNAs.

### Prediction of miRNA Association With CircRNA and Their Target mRNAs

**FIGURE 5:**
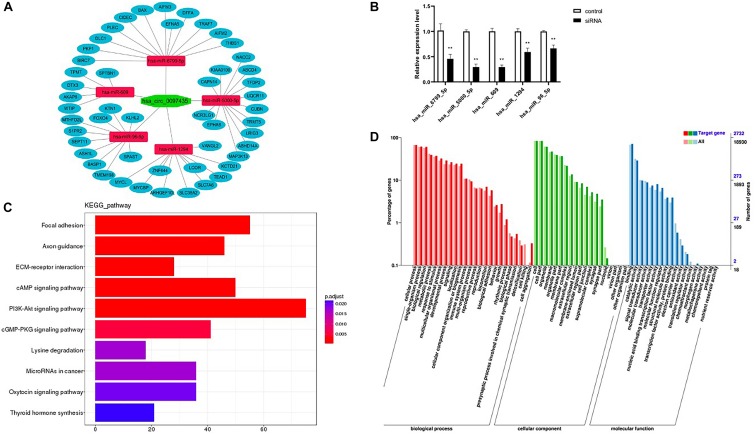
Prediction of miRNA association with circRNA and their target mRNAs. **(A)** The possible interaction of hsa_circ_0097435, miRNAs and mRNAs. **(B)** Compared with the negative-control group, 5 miRNAs upon circ_0097435 silencing were downregulated, including hsa_miR_6799_5P (**p < 0.01), hsa_miR_5000_5P (**p < 0.01), hsa_miR_609 (**p < 0.01), hsa_miR_1294 (**p < 0.01), and hsa_miR_96_5P (**p < 0.01). **(C)** KEGG classification statistics of the mRNAs targeted by the miRNAs associated with hsa_circ_0097435. The abscissa represents the number of genes, the ordinate represents the pathway, and the color of the column represents the corrected *p*-value. **(D)** GO function classification statistics of the mRNAs targeted by the miRNAs associated with hsa_circ_0097435. The abscissa shows the GO classification, the left ordinate shows the percentage of the number of genes, and the right ordinate shows the number of genes.

Based on the sequence information of known miRNAs, miRanda (Supplementary Table S7) and TargetScan (Supplementary Table S8) were used to predict target genes. Intersection target genes of two software and target genes related to cardiac function were selected (Figure 5A and Supplementary Table S9). Then, check the levels of the miRNAs upon hsa_circ_0097435 silencing (Figure 5B). The intersection target genes of miRanda and TargetScan were used for GO analysis and KEGG analysis. The GO annotation system generates a directed acyclic diagram containing three main branches, namely, Biological Processes, Molecular Functions, and Cellular Components. As shown in Figure 5D, GO functions were found out by GO enrichment analysis using hypergeometric testing, and these functions were significantly enriched compared to the entire genome background. In organisms, different gene products coordinate to perform biological functions. Pathway-annotation analysis of the mRNAs targeted by the miRNAs associated with hsa_circ_0097435 is helpful to further interpret the functions of genes. Ten pathways connected with the functions of the mRNAs targeted by the miRNAs associated with hsa_circ_0097435 were defined, based on the KEGG analysis (Figure 5C).

## Discussion

HF is becoming an increasingly prevalent epidemic. Due to the aging of the population and medical advances, increasing number of people are diagnosed with HF and their survival time has been extended, although the mortality rate remains high. As a blood biomarker for HF-related, high-frequency diagnosis and prognosis, BNP is not applicable to all types of HF patients. Another blood biomarker or a group of blood biomarkers is needed to better diagnose and treat HF and predict prognosis. Mounting evidence suggests that HF is mainly caused by genetic variations, that HF has a complex genetic basis (Creemers et al., 2011; Dorn, 2011), and that many biological molecules can serve as potential biomarkers (Maisel and Choudhary, 2012; Wang et al., 2014) or therapeutic targets (Tamargo and Lopez-Sendon, 2011). However, the molecular mechanisms that cause HF are largely unknown. CircRNAs represent a kind of non-coding RNA molecule without a 5′ end cap or 3′ end poly(A) tail, which forms a ring structure with covalent bonds. Its closed ring structure is not easily degraded by exonuclease RNase R, making it more stable than linear RNA. Therefore, circRNAs molecules are potential biomarkers for future HF diagnosis.

NGS was used to screen for differentially expressed circRNAs associated with HF. Fifty-six differentially expressed circRNAs were identified, 29 of which were up-regulated and 27 were down-regulated. To our knowledge, this is the first report describing circRNA-expression profiles in peripheral blood samples from HF patients. Our results can enrich the understanding of HF pathogenesis and provide a theoretical basis for in-depth discussions on the roles of circRNAs in HF. To further verify the up-regulated circRNAs, we increased the sample size and selected 6 circRNAs for qPCR verification. Among the six selected circRNAs, hsa_circ_0097435 (*p* < 0.01) was significantly more abundant in the HF group than in the NC group. The circular structure of hsa_circ_0097435 was verified by Sanger sequencing and a divergent-primer strategy. Further study showed that the hsa_circ_0097435 levels in exosomes from patients with HF was significantly higher (*p* < 0.05) than those from normal volunteers. Based on these experiments, we postulate that most hsa_circ_0097435 is encapsulated in exosomes, which otherwise could be decomposed by the large amount of RNase present in plasmas. This postulate is the same as previous studies found that circRNAs may be encapsulated in exosomes (Li Y. et al., 2015; Zheng et al., 2016).

To understand the role of hsa_circ_0097435 in cardiomyocyte apoptosis, we further examined its expression in DOX-treated cardiomyocytes. Hsa_circ_0097435 was the highest at 12 h after DOX treatment. To verify whether this increase was related to myocardial apoptosis, we conducted a series of validation analyses using loss-of-function and gain-of-function experiments. Hsa_circ_0097435 overexpression promoted cardiomyocyte apoptosis, and silencing hsa_circ_0097435 expression inhibited cardiomyocyte apoptosis. CircRNAs play important regulatory roles in diseases by interacting with disease-related miRNAs. To investigate the mechanism of action between hsa_circ_0097435 and miRNAs, further studies were carried out. In the RNA-pulldown assays, we identified 5 miRNAs that were significantly pulled down following hsa_circ_0097435. In addition, the AGO2 protein was significantly pulled down by hsa_circ_0097435. Immunoprecipitation of AGO2 from AC16 cells showed that the AGO2 protein could directly bind to miRNAs. Among the 4 selected miRNAs, the levels of hsa_miR_6799_5P (*p* < 0.01), hsa_miR_5000_5P (*p* < 0.01), hsa_miR_609 (*p* < 0.01), and hsa_miR_1294 (*p* < 0.01) were significantly higher in the AGO2 group than in the IgG group. Therefore, we predicted that hsa_circ_0097435 could bind to miRNAs through the AGO2 protein and act as a sponge for multiple miRNAs.

To the best of our knowledge, our results provide the first overview of circRNA-expression profiles in HF and the association between hsa_circ_0097435 and multiple miRNAs in HF. Our experiments showed that hsa_circ_0097435 played a crucial role in HF occurrence and development. Based on the hsa_circ_0097435-expression levels observed in exosomes, we speculate that most hsa_circ_0097435 was encapsulated in exosomes. Further functional studies demonstrated that after DOX treatment, myocardial apoptosis increased, and hsa_circ_0097435 could promote apoptosis. In addition, RNA-pulldown experiments and AGO2-immunoprecipitation experiments revealed the potential role of hsa_circ_0097435 in HF via sponging multiple miRNAs. In conclusion, hsa_circ_0097435 can be used as a blood biomarker, and the results further reveal a new pathway that regulates myocardial cell injury.

This study has several limitations. The regulation of hsa_circ_0097435 binding site with miRNA and indirect regulation of the expression of downstream target genes of miRNA become the focus of further research. Experimental identification and characterization of their associated molecules, such as miRNAs or proteins, are suggested in the future. Another limitation of this study is that the effect of hsa_circ_0097435 in exosomes on HF and its mechanism have not yet been determined.

## Data Availability Statement

RNA sequencing data was deposited in the NCBI Sequence Read Archive (BioProject: PRJNA574863).

## Ethics Statement

The studies involving human participants were reviewed and approved by Ethics Committee of affiliated hospital of Qingdao university. The patients/participants provided their written informed consent to participate in this study. Written informed consent was obtained from the individual(s) for the publication of any potentially identifiable images or data included in this article.

## Author Contributions

JH and LZ analyzed the sequencing results of circRNA, completed cell experiment, and wrote this manuscript. LH, HY, FX, BY, RZ, and YZ completed clinical sample collection. YA conceived and designed the research, and revised the manuscript.

## Conflict of Interest

The authors declare that the research was conducted in the absence of any commercial or financial relationships that could be construed as a potential conflict of interest.

## Footnotes

1 http://www.genome.jp/kegg/
