# Supplementary figures and images for: Circular RNA-Expression Profiling Reveals a Potential Role of Hsa_circ_0097435 in Heart Failure via Sponging Multiple MicroRNAs
Source: Front Genet. 2020 Mar 10;11:212. doi: 10.3389/fgene.2020.00212 (PMC7076158; doi:10.3389/fgene.2020.00212)

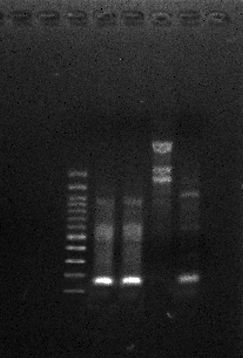

Supplement: Supplementary file 1 [file Image_1.TIF]

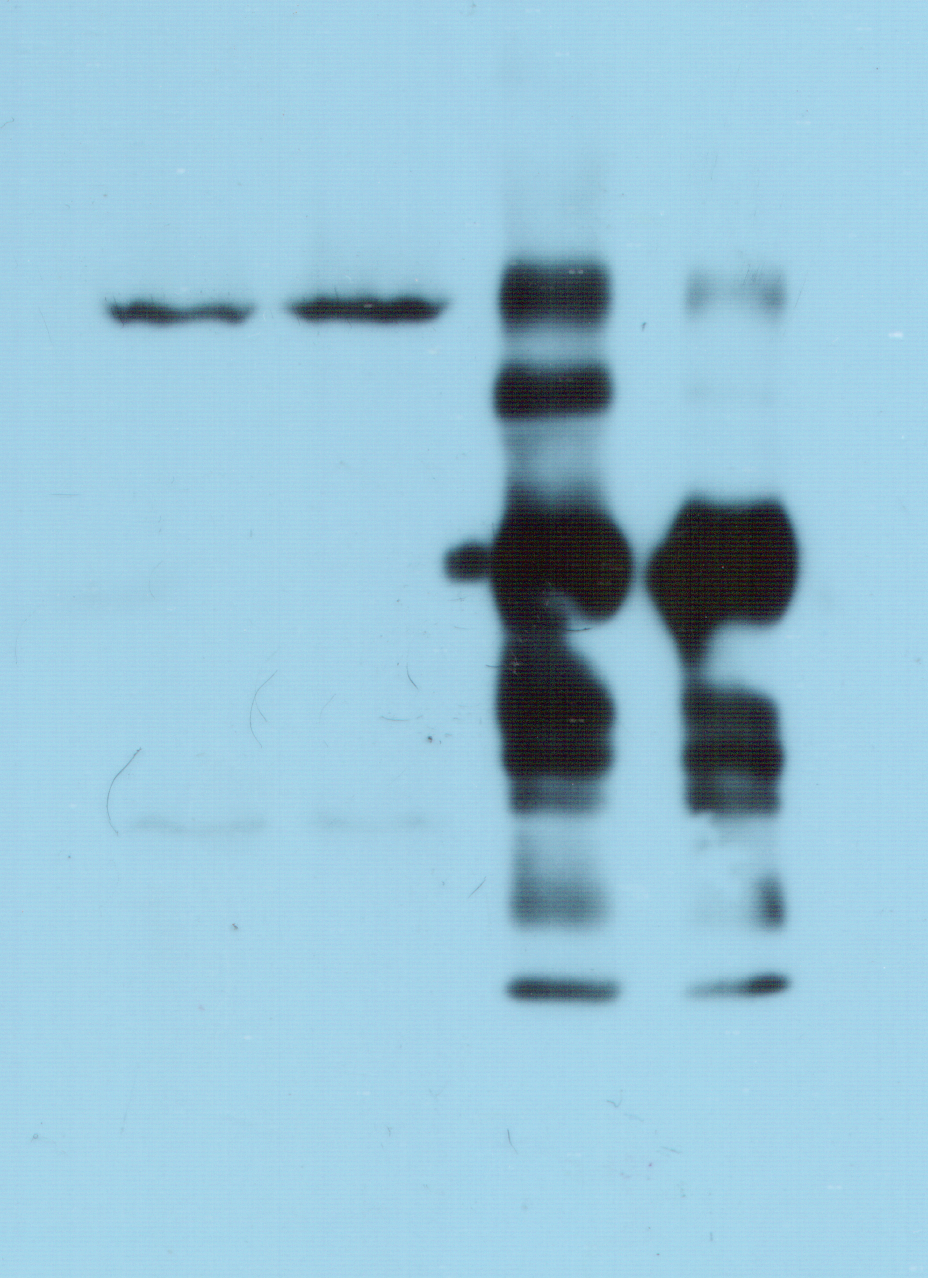

Supplement: Supplementary file 2 [file Image_2.TIF]
