# Supplementary material for: Circular RNA-Expression Profiling Reveals a Potential Role of Hsa_circ_0097435 in Heart Failure via Sponging Multiple MicroRNAs
Source: Front Genet. 2020 Mar 10;11:212. doi: 10.3389/fgene.2020.00212 (PMC7076158; doi:10.3389/fgene.2020.00212)
Supplement: Supplementary file 6 [file Table_4.DOCX]

**Table 4 Sequences of primers used in this study**

| CircRNA | Primer name | Primer sequence (5′–3′) |
| --- | --- | --- |
| hsa_circ_0097435 | hsa_circ_0097435-F | TTTTGAACTTGTGATGCTGACTTG |
|  | hsa_circ_0097435-R | CTCCTCGTTCCGGCAGTACA |
| hsa_circ_0040414 | hsa_circ_0040414-F | CATATGAAAGTGGATTATGGGAGTGA |
|  | hsa_circ_0040414-R | CCCAAACCTGAGAACCAACAC |
| hsa_circ_0001312 | hsa_circ_0001312-F | GGAGGAAAGAGCTAGGTCAATATAGTCT |
|  | hsa_circ_0001312-R | TGTTTAGATGTCTCCTGATGTTCATTT |
| hsa_circ_0005158 | hsa_circ_0005158-F | GCTAAAAGACAGATCAGCAATCGA |
|  | hsa_circ_0005158-R | GCCCGCCTGGGTCTCT |
| hsa_circ_0029696 | hsa_circ_0029696-F | GCAGTTGGAAAAAAGTGACTTTGAA |
|  | hsa_circ_0029696-R | GCCTTTATGAAATCAATGCCTTCTT |
| hsa_circ_0099476 | hsa_circ_0099476-F | AAGAGTGTGATAGGATCGAAAATTAACA |
|  | hsa_circ_0099476-R | CAGTCACCAAAACAATGCACTTC |
