# Supplementary material for: Circular RNA-Expression Profiling Reveals a Potential Role of Hsa_circ_0097435 in Heart Failure via Sponging Multiple MicroRNAs
Source: Front Genet. 2020 Mar 10;11:212. doi: 10.3389/fgene.2020.00212 (PMC7076158; doi:10.3389/fgene.2020.00212)
Supplement: Supplementary file 8 [file Table_6.DOCX]

| CircRNA | MiRNA | TargetScan | MiRanda | RNAhybrid | Binding site |
| --- | --- | --- | --- | --- | --- |
| Hsa_circ_0097435 | Hsa_miR_609 | 1 | 1 | 0 | 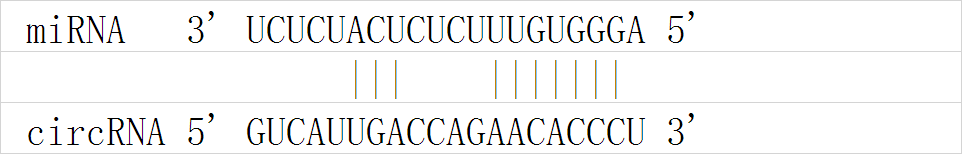 |
| Hsa_circ_0097435 | Hsa_miR_1294 | 1 | 1 | 0 | 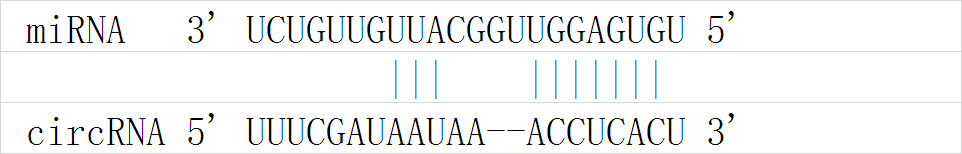 |
| Hsa_circ_0097435 | Hsa_miR_6799_5P | 0 | 1 | 1 | 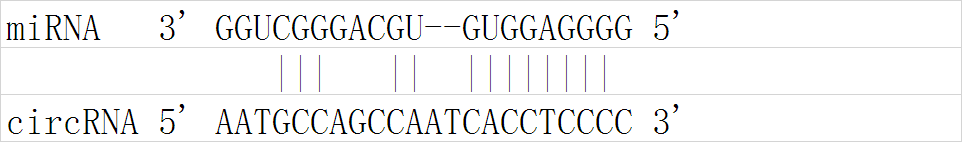 |
| Hsa_circ_0097435 | Hsa_miR_5000_5P | 0 | 1 | 1 | 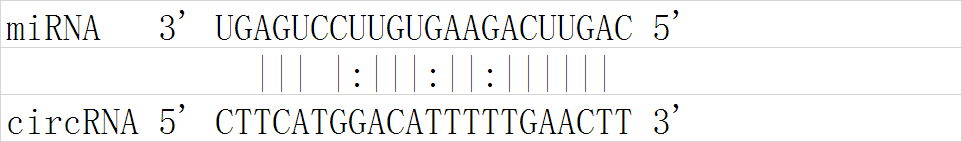 |
| Hsa_circ_0097435 | Hsa_miR_96_5P | 0 | 1 | 1 | 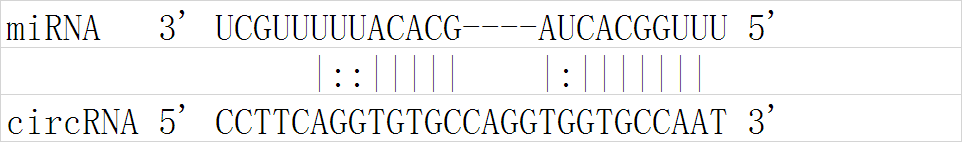 |

**Table 6 Prediction of miRNAs associated with hsa_circ_0097435 and their binding sites.**
