# Supplementary material for: Circular RNA-Expression Profiling Reveals a Potential Role of Hsa_circ_0097435 in Heart Failure via Sponging Multiple MicroRNAs
Source: Front Genet. 2020 Mar 10;11:212. doi: 10.3389/fgene.2020.00212 (PMC7076158; doi:10.3389/fgene.2020.00212)
Supplement: Supplementary file 12 [file Data_Sheet_1.DOCX]

**Explanation of Supplementary Figures and Tables**

**Supplementary Image 1 Validate circular RNA.** Use of the divergent-primer strategy to verify the circular structure of hsa_circ_0097435.

**Supplementary Image 2 WB analysis of pulldown products.** The AGO2 protein was significantly pulled down by hsa_circ_0097435. The amount of AGO2 protein in the C97435-MS2 pulldown group was significantly higher than in the C97435 pulldown group.

**Supplementary Table 1 CircRNA prediction using CIRI.** circRNA_ID: circRNA name; chr: the chromosome; circRNA_start: start position; circRNA_end: end position; #junction_reads: number of junction reads; SM_MS_SMS: CIGAR; #non_junction_reads: number of non-junction reads; junction_reads_ratio: junction_reads / (non_junction_reads / 2 + junction_reads); circRNA_type: circRNA type; gene_id: Source gene ID; strand: direction; junction_reads_ID: ID of junction reads (sequencing clean reads ID).

**Supplementary Table 2 CircRNA prediction using find_circ.** chrom: chromosome name; start: left cut site (starting at 0); end: right cut site (starting with 0); name: circular RNA name; n_reads: number of reads supporting the junction (BED'score ') |-strand: +/-; n_uniq: number of unique reads that support junction; uniq_bridges: Anchor compares the number of unique reads; best_qual_left: Support the best anchor comparison score for left-clip junction; best_qual_right: support the best anchor comparison score for right-cut junction; tissues: sample names, separated by commas; tiss_counts: the number of reads corresponding to each sample, separated by commas; edits: Number of mismatches during anchor extension; anchor_overlap: the number of nucleotides in the breakpoint within an anchor point; breakpoints: break the number of reads in flanking GT / AG mode; signal: flanking nucleotide shear signal (GT / AG); strandmatch: 'MATCH', 'MISMATCH' or 'NA' (non-chain specific analysis); category: Keywords describing junction.

**Supplementary Table 3 CircRNAs with significant differences in expression were identified.**

#Gene: Difference circRNA gene ID; ID: difference circRNA ID; * _Count: sample expression; * _SRPBM: spliced reads per billionmapping; Pvalue: difference Pvalue; log2FC: difference log2FC value; regulated: difference up / down.

**Supplementary Table 4 Sequences of primers used in this study.** Primer sequences of hsa_circ_0097435, hsa_circ_0099476, hsa_circ_0001312, hsa_circ_0005158, hsa_circ_0029696, and hsa_circ_0040414 were in the table.

**Supplementary Table 5 MiRNA predictions using CircInteractome, miRanda and RNAhybrid.** Based on the gene-sequence information of hsa_circ_0097435, miRNA predictions were performed using CircInteractome, miRanda and RNAhybrid.

**Supplementary Table 6 Prediction of miRNAs associated with hsa_circ_0097435 and their binding sites.** 5 miRNAs predicted by at least two softwares including TargetScan, miRanda and RNAhybrid were selected.

**Supplementary Table 7 Target gene prediction using miRanda.** MiRanda was used to predict the target genes of hsa_miR_6799_5P, hsa_miR_5000_5P, hsa_miR_609, hsa_miR_1294, and hsa_miR_96_5P.

**Supplementary Table 8 Target gene prediction using TargetScan.** TargetScan was used to predict the target genes of hsa_miR_6799_5P, hsa_miR_5000_5P, hsa_miR_609, hsa_miR_1294, and hsa_miR_96_5P.

**Supplementary Table 9 Target genes related to cardiac function were selected.** Target genes is the intersection of target genes predicted by the two softwares of miRanda and TargetScan.
